# Evaluation of occupational therapy in persons with COVID-19: a pre-post observational cohort study

**DOI:** 10.1136/bmjopen-2024-089083

**Published:** 2024-11-02

**Authors:** Edith H C Cup, Janneke W B Fleuren-Lemmers, Theodora J Wassink, Lucelle A W van de Ven-Stevens, Philip J van der Wees, Johanna G Kalf, Maud J L Graff

**Affiliations:** 1 Rehabilitation, Radboud University Medical Center, Nijmegen, Netherlands; 2 Community Occupational Therapy Practice JIPA, Wijchen, Netherlands; 3 Dutch Association for Occupational Therapy, Ergotherapie Nederland, Utrecht, Netherlands; 4 IQ Health, Radboud University Medical Center, Nijmegen, Netherlands

**Keywords:** COVID-19, Fatigue, Cognition, Primary Health Care, Patient Reported Outcome Measures, Observational Study

## Abstract

**Abstract:**

**Background:**

Persons with COVID‐19 may experience limitations in daily functioning and can be referred to occupational therapy.

**Objectives:**

1. To evaluate changes in daily functioning, cognitive complaints, fatigue and self-management of persons with COVID-19 who received occupational therapy in primary care.
2. To get insights in the volume and duration of occupational therapy.

**Design:**

A pre-post observational cohort study from October 2020 until April 2021.

**Setting:**

Fifty-eight occupational therapy practices in primary care throughout the Netherlands participated with 68 occupational therapists.

**Participants:**

228 adults (≥18 years) with COVID-19, referred to occupational therapy, gave informed consent and participated in the pre-post evaluation. The mean age was 49 years (SD 13) and 79% of the patients was female. The most frequently reported complaints included fatigue and cognitive complaints.

**Interventions:**

Occupational therapy using Dutch guidelines for occupational therapy in clients with COVID-19.

**Outcome measures:**

Performance and satisfaction with performance using the Canadian Occupational Performance Measure (COPM); the impact of Cognitive Complaints on Participation (CoCo-P); and daily activities, self-management and perceived contribution of occupational therapy using the Patient Reported Outcome Measure for Occupational Therapy (PROM-OT).

**Results:**

COPM-performance score improved with a mean difference of 2.9 points (95% CI 2.7 to 3.2), and COPM-satisfaction score improved with 3.2 points (95% CI 2.9 to 3.5). CoCo-P score improved with a mean difference of 20.9 points (95% CI from 14.4 to 27.4), and PROM-OT improved with 42.8 points (95% CI from 40.2 to 45.4). Participants received a median of seven sessions of occupational therapy (IQR 5–10) with a median duration of 18 weeks (IQR 12–25). They valued the contribution of occupational therapy to their improved functioning with a mean score of 8 (SD 1.4) and recommended this to others with a mean score of 9 (SD 1.2).

**Conclusions:**

Persons with COVID-19 who received occupational therapy in primary care improved significantly in daily functioning and highly valued occupational therapy.

**Strengths and limitations of this study**

- This pragmatic observational cohort study took place during the COVID-19 pandemic with many restrictions and no possibility to perform a randomised controlled trial.
- This study has high ecological validity with 68 participating occupational therapists from 58 occupational therapy practices in primary care throughout the Netherlands who shared their treatment data.
- The occupational therapy-specific outcome measures fit well with the most reported complaints (fatigue and cognitive complaints), limitations in daily activities, restrictions in participation and self-management.
- Although practical guidelines for occupational therapy in clients with COVID-19 were available, we do not know what the content was of the occupational therapy interventions provided.

## Introduction

 The COVID‐19 pandemic, caused by SARS-CoV-2, has caused a variety of functional complaints resulting in participation problems by a lot of persons worldwide. About 90% of persons experienced fatigue, cognitive problems including difficulties with concentration, memory and processing stimuli, as well as loss of stamina and shortage of breath on exertion, even after 1 year.^1^ Over 90% experienced problems performing their daily activities with large consequences for participation in all life domains including self-care, family life, productivity and leisure. Many persons were referred to allied healthcare in primary care, including occupational therapy,^1^,^3^ which aims to support people in improving their daily functioning, participation and self-management.

A survey among occupational therapists (n=550) from 69 countries on the delivery of occupational therapy services to persons with COVID-19^4^ indicated that occupational therapists provided a range of interventions, aligned with the evidence-based WHO COVID-19/PCC Guidelines^5^ with interventions aiming at participation in daily activities, activity tolerance, fatigue management, cognitive functioning and self-management skills. The survey identified a strong role for occupational therapists, who generally rated their services as effective. Recommendations included the use of research evidence to guide occupational therapy services for persons having had COVID-19, as well as the creation of quality service standards and accessibility and availability of occupational therapy.^4^

Already in the beginning of the pandemic, the Dutch association of occupational therapy (Ergotherapie Nederland) together with occupational therapists and occupational therapy researchers have developed practical guidelines^6 7^ and supported its use with webinars for occupational therapists. These guidelines included a combination of assessments and interventions for managing the impact of the most limiting symptoms and complaints on daily activities and participation, with special attention for participation in work.^6 7^

During the pandemic in July 2020, the Dutch Ministry of Health, Welfare and Sports instated a temporary regulation in primary allied healthcare (dietetics, occupational therapy, physical therapy/exercise therapy and speech and language therapy) to facilitate the treatment of persons recovering from COVID-19 and to promote research. This regulation enabled the reimbursement of primary allied healthcare for every patient from basic health insurance coverage. With a referral from a general practitioner or medical specialist, primary allied healthcare treatment was reimbursed for a period of 6 months. There was a maximum of 10 hours of occupational therapy within those 6 months. If recovery during this period was insufficient, an extension by a second 6 month period was possible on referral by a medical specialist. As there was not yet evidence for allied healthcare in this new diagnosis, willingness to participate in research was conditional to obtain the reimbursement from basic health insurance coverage.

Most allied health professions (physical therapy, exercise therapy, speech and language therapy and dietetics) in the Netherlands have national data registries and can obtain insight in patient characteristics and volume of allied healthcare provided.^8^ Until now this is not the case for occupational therapy. Therefore, occupational therapists and their clients with COVID-19 were encouraged to participate in nationwide research to get insights in the characteristics of persons with COVID-19 that received occupational therapy and in the volume of care provided and in the changes in daily functioning of persons receiving of occupational therapy. The Dutch association of occupational therapy (Ergotherapie Nederland) initiated a survey among occupational therapists to get insights in the number of patients that were referred and whether or not the occupational therapists made use of the temporary regulation for the reimbursement.^9^ The current research was initiated by the occupational therapy research group of Radboudumc in collaboration with Ergotherapie Nederland.

The research aims of current study were to evaluate the changes in daily functioning and participation of persons with COVID-19 who received occupational therapy in primary care and to obtain insights in the volume of occupational therapy delivered.

Primary research question was:

- What are the changes in daily functioning, participation, cognitive complaints and fatigue impacting participation and in self-management of persons with COVID-19 who received occupational therapy in primary care?

Secondary research questions included:

- What are the characteristics and complaints of the persons with COVID-19 referred to occupational therapy?
- What was the volume and duration of occupational therapy provided and how many occupational therapy practices and occupational therapists thereof participated?
- How did the participants perceive the contribution of occupational therapy to the ability to perform daily activities?

## Methods

### Study design and setting

An observational cohort study with pre-post measurements was carried out evaluating the changes in daily functioning and participation of persons with COVID-19 who received occupational therapy in primary care in the Netherlands. The inclusion period was between October 2020 and April 2021.

### Participants

Adult persons (aged ≥18 years) with complaints following COVID-19 were eligible for inclusion if they were referred to and started with occupational therapy in primary care, if they had given informed consent and if they were able to complete self-reported questionnaires. The occupational therapist had to send the registration form with general characteristics and occupational specific outcome measures before and after occupational therapy.

Persons were excluded from analysis when only baseline characteristics and baseline data were sent and no data on outcome measures were sent after having had occupational therapy.

### Data collection

Through newsletters and social media posts, the Dutch association for occupational therapy (Ergotherapie Nederland) asked Dutch occupational therapists to participate in the study and provided them with the information about the study and provided access to registration forms and measurement instruments. The occupational therapists provided their clients with COVID-19 with verbal and written information about the study. After they had signed the informed consent form, the occupational therapists sent this by secure email to the researchers of the Radboud university medical centre (Radboudumc). They also sent the registration form for reporting baseline characteristics (sex, age, diagnosis) and the main complaints of the persons having had COVID-19. Also the priorities in daily occupations and scores for performance and satisfaction of the Canadian Occupational Performance Measure (see outcome measures) were recorded on the registration form. The registration form also included the number of sessions, starting date and end date of occupational therapy treatment. Together with the registration forms the questionnaires (CoCo-P and PROM-OT, see outcome measures) were sent to Radboudumc via secure email. All forms were stored according to the Secure Data Management regulations of the Radboudumc.

### Occupational therapy intervention

Practical guidelines for occupational therapy following COVID-19 were available through the website of the Dutch association for occupational therapy.^6 7^ The association also offered webinars for occupational therapists to support its use. However, occupational therapists were not asked to use specific interventions.

### Baseline characteristics

The baseline characteristics of the persons having had COVID-19 included age and sex and their most important complaints in their own words in open text. These complaints were divided into the following categories by JWBF-L following discussion with EHCC: fatigue, cognitive complaints, respiratory complaints, physical complaints, pain, autonomic dysregulation and psychological complaints. These categories were decided among two researchers, the categorisation of the complaints was done by one researcher (JWBF-L).

### Outcome measures

#### Performance and satisfaction with personal participation priorities

The Canadian Occupational Performance Measure (COPM) is a semi-structured interview to identify and prioritise problems in daily activities and participation in the areas of self-care, productivity and leisure.^10 11^ Three to five priorities are scored on a ten-point rating scale from 1 to 10 for performance and for satisfaction with performance; higher scores indicate better performance and more satisfaction. The COPM has been used worldwide by occupational therapists and researchers and has shown good reliability and validity in different client groups.^10 12 13^ In various studies, a difference of two points was defined as a clinically significant difference.^13^,^15^

#### Cognitive complaints and fatigue impacting participation

The Cognitive Complaints–Participation (CoCo-P) is a patient (and relative)-reported outcome measure that evaluates cognitive complaints related to memory, attention and executive functioning during participation in daily activities.^16^ Ten participation domains are distinguished: work, leisure, travel, driving, social activities, family, medicines, finances, shopping and cooking. Each domain contains two or more statements and is scored on a three-point rating scale, whereby 0 indicates ‘independent without effort’ and 3 indicates ‘not possible’. It includes a fourth option ‘ not applicable’, as some activities (eg, driving a car, use of medication) are not applicable for some patients. A total sum score is computed based on all items. The scores are converted to a 0–100 scale with the formula: (Mean score/3(maximum score per item)) ×100. Higher scores indicate more cognitive complaints during daily activities or more dependency in participation. At the end of each participation domain, a visual analogue scale (0–100 mm) is scored for fatigue, the higher the scores, the more fatiguing participation in that domain is. A total fatigue score is calculated by summing all fatigue scores (0–100). The CoCo-P was originally developed together with and for persons with cognitive complaints resulting from acquired brain injury. The researchers contacted the developers and received approval to use the CoCo-P in this research with persons with complaints following COVID-19. Although there is a CoCo-P version for persons with cognitive complaints and a CoCo-P version for a relative, in this article, only the outcome on the CoCo-P completed by persons with COVID-19 is reported.

#### Daily activities, self-management, management by relatives and contribution of OT

The Patient-Reported Outcome Measure for Occupational Therapy (PROM-OT, in Dutch: PRO-Ergo) is an occupational therapy-specific patient-reported outcome measure.^17^ It consists of 13 statements, of which the first 11 are scored before and after an occupational therapy intervention on an 11-point rating scale from 0 (completely disagree) to 10 (completely agree), the higher the scores, the better the daily life management. The last two statements are about the contribution of OT to the ability to perform daily activities and whether one would recommend occupational therapy to others with similar complaints. Reliability, validity and responsiveness of the PROM-OT are good.^17^,^19^ Factor analysis revealed three factors: daily activities, self-management and management by relatives, which are presented as the PROM-OT subscales.^18^

### Statistical analysis

Descriptive statistics were used to describe the patient characteristics as well as the volume of occupational therapy, using means and SD for continuous variables and medians and IQRs for ordinal variables. To evaluate change in the outcomes of the COPM, CoCo-P and PROM-OT, paired sample t-tests were used. Mean differences and 95% CIs are presented. P values of <0.05 were considered to be statistically significant, and Cohen’s d effect size was interpreted according to the empirically derived effect size guidelines for multicomponent rehabilitation^20^: from 0.14 to 0.31 is considered a small effect size; 0.31 to 0.55 a medium effect size and 0.55 or more is considered a large effect size. All data were analysed using SPSS statistics 25 (IBM).

## Results

### Participants

Informed consent was obtained by the occupational therapists in primary care from 316 persons with complaints following COVID-19. Data on volume of occupational therapy and outcomes pre- and post-intervention were obtained from 228 persons and are presented in this article. There were 180 women (79%) and 47 men (20%) and one person did not respond to this question. The mean age was 49 years (SD13). The most reported complaints were fatigue and cognitive problems. From the 228 persons, 200 participants had reported their most important complaints (table 1).

**Table 1:** Complaints of persons following COVID-19 (n=200)

Complaints	N (%)
Fatigue	178 (89 %)
Cognitive complaints	124 (62 %)
Respiratory problems	39 (19.5 %)
Physical complaints	32 (16 %)
Pain	29 (14.5 %)
Autonomic dysregulation	13 (6.5 %)
Psychological complaints	2 (1 %)

N, the number of participants with available data

### Occupational therapy provided in primary care

Initially 92 occupational therapists from 75 occupational therapy practices in primary care throughout the Netherlands sent informed consent forms of 316 persons with COVID-19. From 68 occupational therapists working in 58 practices, information on volume and outcome pre- and post-occupational therapy was obtained of 228 persons. They received a median number of seven sessions (IQR 5–10) with a median duration of 18 weeks (IQR 12–25).

### Performance and satisfaction with personal participation priorities

In 213 participants the COPM was administered at the start (T0) as well as at the end of treatment (T1). The individualised priorities that were identified included a wide variety of activities from all three areas of self-care, productivity and leisure. The mean COPM performance score improved from 4 (SD 1.5) at T0 to 7 (SD 1.6) at T1, with a mean difference of 2.9 points (95% CI 2.7 to 3.2). The mean COPM satisfaction score improved from 3.9 (SD 1.6) at T0 to 7.1 (SD 1.6) at T1, with a mean difference of 3.2 points (95% CI 2.9 to 3.5). Effect sizes were 1.6 for performance and 1.5 for satisfaction (table 2). Of all participants, 81% had clinically relevant improvement of 2 points or more on the COPM performance scale and 77% on the satisfaction scale.

**Table 2:** Means and SD in the occupational therapy specific outcome measures at T0 and T1.

Outcome measures	N T0	Mean (SD) at T0	Mean (SD) at T1	Mean difference and 95% CI	P value	Cohen’s d
**COPM**						
Performance score	213	4 (1.5)	7.0 (1.6)	2.9 (2.7 to 3.2)	<0.001	1.6
Satisfaction score	211	3.9 (1.8)	7.1 (1.6)	3.2 (2.9 to 3.5)	<0.001	1.5
**CoCo-P participation domains (higher scores indicate more dependency**)						
Total CoCo-P score	103	47.2 (36.4)	26.2 (14.1)	20.9 (14.4 to 27.4)	<0.001	0.6
Work/education	93	42.0 (21.7)	25.2 (18.7)	16.8 (12.4 to 21.1)	<0.001	0.8
Leisure activities	101	34.9 (23.2)	16.9 (18.7)	17.9 (13.0 to 22.9)	<0.001	0.7
Travel	41	22.0 (29.2)	15.9 (24.4)	6.1 (-4.2 to 16.4)	0.239	0.2
Driving	96	14.8 (20.9)	8.0 (18.0)	6.7 (2.1 to 11.3)	0.005	0.3
Social contacts	107	24.5 (21.1)	15.6 (17.2)	8.9 (4.6 to 13.1)	<0.001	0.4
Family life	102	33.1 (24.5)	17.8 (19.5)	15.4 (10.7 to 20.0)	<0.001	0.6
Use of medication	69	11.3 (18.5)	5.6 (12.5)	5.8 (1.8 to 9.8)	0.005	0.4
Finances	100	8 (17.6)	5 (13.3)	3.0 (0.5 to 5.5)	0.17	0.2
Grocery shopping	79	24.8 (22.8)	11.4 (14.7)	13.4 (8.8 to 17.9	<0.001	0.7
Cooking	100	20.4 (17.3)	10.9 (12.7)	9.4 (6.1 to 12.8)	<0.001	0.6
**CoCo-P fatigue (higher scores indicate more fatigue**)						
Total Fatigue score	25	51.0 (19.7)	36.1 (23.6)	14.9 (6.7 to 23.3)	0.01	0.7
Work/education	86	76.9 (17.3)	52.2 (26.9)	24.8 (18.6 to 30.9)	<0.001	0.9
Leisure activities	93	59.9 (24.8)	40.8 (28.4)	19.1 (12.5 to 25.6)	<0.001	0.6
Travel	46	54.8 (24.5)	45.5 (30.9)	9.3 (−0.6 to 19.1)	0.064	0.3
Driving	90	55.5 (27.8)	38.9 (30.1)	16.6 (9.7 to 23.5)	<0.001	0.5
Social contacts	95	53.8 (25.5)	37.1 (26.9)	16.7 (11.3 to 22.2)	<0.001	0.6
Family life	94	52.5 (22.4)	33.9 (25.2)	18.6 (13.0 to 24.3)	<0.001	0.7
Use of medication	66	16.5 (20.6)	13.6 (16.4)	2.9 (-1.5 to 7.3)	0.196	0.2
Finances	95	24.3 (24.7)	17.9 (20.4)	6.3 (1.7 to 10.9)	0.007	0.3
Grocery shopping	95	63.4 (25.1)	38.6 (29.8)	24.8 (18.5 to 31.2)	<0.001	0.8
Cooking	95	55.8 (27.2)	35.0 (28.3)	20.8 (14.9 to 26.7)	<0.001	0.7
**PROM-OT (higher scores indicate better performance**)						
Total PROM-OT score	180	56.4 (15.3)	99.2 (14.6)	42.8 (40.2 to 45.4)	<0.001	2.4
Daily activities	189	21.33 (8.3)	35.4 (7.6)	14.1 (12.7 to 15.4)	<0.001	1.5
Self-management	191	20.9 (6.5)	30.1 (4.7)	9.3 (8.3 to 10.2)	<0.001	1.4
Management relatives	188	14.2 (3.8)	16.2 (2.7)	1.9 (1.4 to 2.5)	<0.001	0.5

CIConfidence intervalCoCo-PCognitive Complaints-ParticipationCohen’s deffect sizeCOPMCanadian Occupational Performance MeasureNthe number of participants with available data; missing data are explained by persons not having completed the itemsPROM-OTPatient Reported Outcome Measure - Occupational TherapyP-valuelevel of significancySDStandard deviationT0, initial assessment, before occupational therapy interventionT1post intervention assessment

### Cognitive complaints and fatigue impacting participation in daily activities

The CoCo-P was completed at the start as well as at the end of occupational therapy in 107 participants. The participation domains work/education, leisure activities and family life had the highest scores, meaning that dependency in these participation domains was most impacted by cognitive complaints. On these domains the improvements were largest. The total score of the CoCo-P improved significantly from 47.2 (SD 36.4) at T0 to 26.2 (SD 14.1) at T1 with a mean difference of 20.9 (95% CI 14.4 to 27.4) and Cohen’s d effect size of 0.6 (table 2).

The level of independence in participation (CoCo-P participation scores) before and after OT improved in all ten domains, with statistically significantly improvements in eight domains. The improvements in the domains travel and finances were not statistically significant (table 2). The CoCo-P fatigue scores also improved in all domains, with statistically significant improvements (p<0.05) in eight domains with Cohens’d effect sizes varying from 0.2 for use of medication to 0.9 for work/education (table 2).

### Patient-Reported Outcome Measure for Occupational Therapy (PROM-OT)

The changes in the total scores and domains of the PROM-OT were evaluated both at the start and at the end of occupational therapy in 189 participants. The total score on the PROM-OT improved significantly from 56.4 (SD 15.3) at T0 to 99.2 (SD 14.6) at T1 with a mean difference of 42.8 (95% CI 40.2 to 45.4). There were significant improvements in all three domains of the PROM-OT and Cohens’d effect sizes varying from 0.5 (management by relatives) to 1.5 (daily activities) and 2.4 for the total PROM-OT score (table 2).

The statements of the PROM-OT about the contribution of occupational therapy to participants’ daily functioning had a mean score of 8 (SD 1.4), and the statement whether one would recommend occupational therapy to others with similar complaints had a mean score of 9 (SD 1.2).

## Discussion

In this study, changes in daily functioning of persons with COVID-19 following occupational therapy in primary care were evaluated. There were significant improvements on all three outcome measures, meaning improvements in performance and satisfaction in daily functioning and participation (COPM), decrease of the impact of cognitive complaints and fatigue on participation (CoCo-P) and improvements in daily activities, self-management and management by relatives (PROM-OT). Persons with COVID-19 very much valued the contribution of occupational therapy and highly recommended this to others with similar complaints. Despite improvements, there were still residual complaints following COVID-19 impacting daily functioning.

### Strengths and weaknesses of the study

This cohort study was the first study evaluating occupational therapy for persons with COVID-19. It was an observational study and not set up as an effectiveness study. This pragmatic study was carried out early in the pandemic in a society with many restrictions and depended on the willingness of occupational therapists and their clients to participate in this study. This raises questions regarding the representativeness and generalisability of this study. Although there was a special regulation for the reimbursement of allied healthcare in primary care for persons with COVID-19, which required participation in research, it has become clear from several nationwide surveys that not all occupational therapists made use of this regulation.^9^ In November 2020, only 29% of the occupational therapists in primary care who had completed the survey made use of this regulation, and in February 2021, this had amounted up to 56%.^9^ These nationwide surveys provided some reference data regarding the number of persons with COVID-19 receiving occupational therapy. In February 2021 there were 106 occupational therapists working in primary care who responded to the survey. Together they had treated 1100 persons with COVID-19 of which 700 patient still received treatment. In comparison with the current study, initially 92 occupational therapists in primary care sent informed consent forms of 316 persons with COVID-19. Ultimately there were complete data sets (before and after occupational therapy) of 228 patients from 68 occupational therapists. We do not know whether the occupational therapists included their full caseload of eligible persons with COVID-19 or not. Our study obviously included a selection of all persons with COVID-19 receiving occupational therapy.

Reasons for not using the special reimbursement regulation were discussed at the yearly occupational therapy conference of 2024 in a workshop on the use of the practical guidelines for occupational therapy for persons with COVID-19.^21^ Occupational therapists revealed two reasons for not using the regulation and not participating in the research: (1) the additional administrative workload involved in an already stressful time during the COVID-19 pandemic with increasing waiting lists for occupational therapy and (2) ethical and moral concerns regarding the requirement to participate in research to receive reimbursement. In the Netherlands persons have access to 10 hours of occupational therapy reimbursed by the health insurance companies anyway, regardless of the diagnosis.

Unfortunately we were unable to monitor the completeness of the data and we have not collected data on reasons for the loss of follow-up or for not completing questionnaires. We can only make assumptions that occupational therapists and the persons with COVID-19 receiving occupational therapy were willing to participate initially, but were unable to send data at the end of treatment due to the lack of time, motivation, energy or other reasons. In the national survey among occupational therapists, more than half of occupational therapists reported that they were unable to do the all treatments and had to take measures and refer patients to colleagues in primary care.^9^

The selected outcome measures fitted well with the most reported complaints (fatigue and cognitive complaints) impacting daily activities and participation and were completed by a large group of participants. The feasibility of evaluating occupational therapy in primary care with these outcome measures appears promising. The outcome measures were patient-reported, and the COPM was administered by the occupational therapists who provided the therapy, meaning that there was no independent assessor collecting the outcome measures, which involved a risk of bias.

An advantage of the administration of the COPM by occupational therapists who send the data was the largest response rate of the COPM compared with the other outcome measures. The other questionnaires were independently completed by persons with COVID-19 who were often very fatigued and may have had cognitive difficulties.

This was the first study evaluating occupational therapy using the PROM-OT as an outcome measure. The response rate of this self-report outcome measure was about 80% (table 2), which was twice as high as the response rate for the CoCo-P, which was about 40%. The length of the CoCo-P (53 items) might have influenced this CoCo-P response rate. In eight out of ten participation domains of the CoCo-P, participants reported that their dependency decreased significantly (p<0.05). The two participation domains that did not show significant improvements were travel and finance. Travelling was hardly allowed during the COVID-19 pandemic due to the restrictive measures and was only completed by 18% of the persons. The participation domain finances were completed by 30% of the participants, who hardly experienced problems in this domain. The mean fatigue score for the different participation domains of the CoCo-P showed a significant reduction in all domains, except for the domains travel, which was hardly allowed and use of medication. The relatively young participants hardly used medication, which was generally not experienced as tiring. Although the CoCo-P was developed with and for persons with acquired brain injury, this outcome measure seemed responsive to changes in current study with persons following COVID-19.^22^

The Dutch occupational therapy association had developed the occupational therapy guidelines for persons following COVID-19 together with occupational therapists in practice,^6 7^ which were available for all occupational therapists from the website.^23^ The use of the guidelines was promoted and webinars on the procedure and outcome measures were offered. As adherence to these guidelines was not evaluated and the content of the occupational therapy interventions was not specifically registered for this study, it is unknown which interventions were applied. However, a survey among occupational therapists by the Dutch association for occupational therapy provided insight which domains were mostly addressed in primary care. These included interventions to manage fatigue (93%), return to work (73%) and management of cognitive complaints (71%).^9^

In the absence of a control group of persons with COVID-19 that did not receive occupational therapy, we do not know which results can be attributed to occupational therapy or to other therapies. In the current study, we did not collect information on concurrent therapies. A subsequent cohort study on allied healthcare for persons with COVID-19 showed that 62% of the patients had received more than one allied health therapy.^24^ Another factor that might have influenced the results includes natural recovery. Also response shift may have occurred as persons with COVID-19 may adapt and self-manage despite their complaints. This process of adaptation might also have been the result of occupational therapy as the goal is to support persons in self-managing life despite complaints.^6 7^

### Comparison to other studies

Our cohort study evaluating occupational therapy for persons with COVID-19 was followed by a nationwide prospective cohort study evaluating allied health recovery care for persons with COVID-19 including physiotherapy, exercise therapy, speech therapy and dietetics, as well as occupational therapy, called the ParaCov study.^2 25^ This study included 1452 persons from 29 March 2021 to 19 June 19 2021. Their recovery was monitored at 3, 6, 9 and 12 months.^2 3 25^ In the ParaCov study, the mean age of the participants was also below 50 and the majority was woman. The main complaints (fatigue and cognitive complaints) were similar, and the main participation goal for occupational therapy was work (Cup *et al*, in preparation). Also other cohort studies with persons following COVID-19 nationally^1 26 27^ and internationally^28 29^ showed similar characteristics. Another agreement was that, despite significant improvements, there were persisting limitations in daily functioning.^3 25 28 30^

In our study a median of seven sessions of occupational therapy was provided. The duration of a sessions is generally 45–60 min,^9^ and the mean duration 18 weeks. The number of sessions is slightly lower compared with other cohort studies evaluating the volume of occupational therapy following COVID-19. In the ParaCov study,^2 25^ 364 persons received occupational therapy, whereby a median number of eight sessions was reported (Cup *et al*, in preparation). In another large Dutch survey (n=8630 respondents with COVID-19), a mean of nine appointments (SD 9) with the occupational therapist was reported.^30^ The Dutch survey among occupational therapists reported that two-thirds of the persons with COVID-19 received 4–10 sessions of occupational therapy and one-third received 10–20 sessions.^9^ More than half of the persons with COVID-19 had a treatment duration of more than 3 months, and nearly 20% exceeded a treatment duration of 6 months.^9^ The large variability in duration of occupational therapy can be explained by differences in severity of COVID-19 and persistence of complaints as well as differences in the impact this has on people’s daily lives and the variety in support needs. As fatigue was the most reported complaint interfering with daily life, occupational therapists give education on managing fatigue and support people in practising fatigue-management skills and strategies.^31 32^ People need time to practice and to integrate self-management strategies in their daily lives and evaluate the experiences with the occupational therapist who has the role of a self-management coach. This might explain a treatment duration of on average 18 weeks, whereby the number of sessions can be low but extended over many weeks.

When our cohort study had started in the fall of 2020, there were waiting lists for occupational therapy, which might have resulted in some under-use of occupational therapy. In the Dutch survey among occupational therapists the creation of waiting lists was mentioned as the most important measure to deal with the large number of persons with COVID-19 being referred to occupational therapy.^9^ The mixed methods study as part of the ParaCov study described that ‘occupational therapists were much less frequently involved and sometimes only late in treatment because of their waiting list’.^33^

In our study, participants were generally very positive about the occupational therapy services and recommended this to others. This is in agreement with the findings in the large national survey among persons with COVID-19, who rated satisfaction with all health services and were generally most satisfied with the occupational therapy services.^30^ Although we do not know which aspects of occupational therapy persons were most satisfied with, we may learn from the mixed methods study aiming to understand how persons with COVID-19, who had received allied healthcare, had dealt with their persistent complaints. In that study the participants reported that a listening ear, support in managing limits and acceptance of building up in small steps were most valuable.^33^ These aspects were derived from the experiences with the whole allied health recovery care including occupational therapy and are aspects that occupational therapists addressed during their interventions with persons with COVID-19.^6 7^

### Implications and future research

We have learnt from this study that many occupational therapists and their clients in primary care are willing to participate in research evaluating occupational therapy in primary care. Although persons with COVID-19 still had residual limitations in daily functioning and participation, there were significant improvements in daily functioning and participation following occupational therapy and people were very satisfied with the contribution of occupational therapy to their ability to manage daily activities and participation. This suggests that it is important to continue to refer to occupational therapy and reimburse occupational therapy for persons with COVID-19 or similar infections in the future.

As in practice persons with COVID-19 often received a combination of allied healthcare professionals, research needs to investigate in what order or in what combination the different therapies can be provided optimally. An interesting initiative is set up in the United Kingdom whereby persons with lived experience of long COVID together with rehabilitation practitioners with (lived) experience will co-design personalised self-management support and evaluate this.^34^ What makes this initiative even more interesting is the strategies to involve harder-to-reach groups from diverse backgrounds.

As occupational therapy, whether or not combined with other allied health interventions, can be considered a complex intervention, its research goes beyond solely evaluating the outcome.^35^ A range of other questions need to be addressed, for example, regarding the content of interventions, its working mechanisms and the critical ingredients. This asks for a mixed methods approach involving diverse stakeholder perspectives. Also, evaluating the effectiveness of OT in a research design with a control group (eg, persons on the waiting list) is recommended.

## Data Availability

Data are available upon reasonable request.

## References

[1] Brus I. SI, Tieleman P, Biere-Rafi S (2022). De invloed van Long COVID op gezondheid, dagelijks leven, werk en zorg. Meerjarig Long Covid onderzoek: rapport jaar 1. C-support.

[2] De Bie RA, Verburg AC, Agasi-Idenburg C (2022). Evaluation of Allied Healthcare in Patients Recovering from Covid-19: Study Protocol and Baseline Data of s National Prospective Cohort Study. JRM.

[3] Slotegraaf AI, Gerards MHG, Verburg AC (2023). Evaluation of Primary Allied Health Care in Patients Recovering From COVID-19 at 6-Month Follow-up: Dutch Nationwide Prospective Cohort Study. JMIR Public Health Surveill.

[4] von Zweck C, Naidoo D, Govender P (2023). Current Practice in Occupational Therapy for COVID-19 and Post-COVID-19 Conditions. Occup Ther Int.

[5] (2022). Clinical Management of COVID-19: Living Guideline.

[6] Wassink D, Ven-Stevens L (2021). Handreiking Ergotherapie Bij COVID-19 Cliënten in de Herstelfase.

[7] Wassink D, Ven-Stevens L (2022). Handreiking Ergotherapie Bij Cliënten Met Het Post-COVID-Syndroom.

[8] Verburg AC, Bie RA, Hoogeboom TJ (2023). Paramedische Herstelzorg Na COVID-19 – Inzicht in Verloop van de Eerstelijns Zorg over de Tijd.

[9] Wassink D, Rouwelaar te B, Ven-Stevens L (2021). Het eerste coronajaar’: ergotherapie in de eerste- en tweede lijn in beeld. Ergother Mag.

[10] Dedding C, Cardol M, Eyssen IC (2004). Validity of the Canadian Occupational Performance Measure: a client-centred outcome measurement. Clin Rehabil.

[11] McColl MA, Law M, Baptiste S (2005). Targeted applications of the Canadian Occupational Performance Measure. Can J Occup Ther.

[12] Eyssen IC, Beelen A, Dedding C (2005). The reproducibility of the Canadian Occupational Performance Measure. Clin Rehabil.

[13] Eyssen IC, Steultjens MPM, Oud TAM (2011). Responsiveness of the Canadian occupational performance measure. J Rehabil Res Dev.

[14] Veenhuizen Y, Cup EHC, Jonker MA (2019). Self-management program improves participation in patients with neuromuscular disease: A randomized controlled trial. Neurology (ECronicon).

[15] McColl MA, Denis CB, Douglas K-L (2023). A Clinically Significant Difference on the COPM: A Review. Can J Occup Ther.

[16] Spreij LA, Sluiter D, Gosselt IK (2021). CoCo - participation: The development and clinical use of a novel inventory measuring cognitive complaints in daily life. Neuropsychol Rehabil.

[17] Arnoldus BE, Dijk S, Hermans Y (2020). De Validiteit van de PRO-Ergo. Ergotherapie Magazine.

[18] Marsman Q (2021). Interne Consistentie En Responsiviteit van de PRO-Ergo.

[19] Ven-Stevens LCE, Wassink D, Satink T (2022). Meten is weten. Steeds meer aandacht voor meten vanuit perspectief van de cliënt: de PRO-ERgo. Ergother Mag.

[20] Kinney AR, Eakman AM, Graham JE (2020). Novel Effect Size Interpretation Guidelines and an Evaluation of Statistical Power in Rehabilitation Research. Arch Phys Med Rehabil.

[21] Cup E Ergotherapie Bij Mensen Na COVID-19. Ergotherapie Jaarcongres 2024; 2024; 1931 Congrescentrum ’s Hertogenbosch.

[22] Heister M (2023). Clinimetric properties of the coco-p in adults with post covid-19 condition: can we really distinguish cognitive functions. master thesis.

[23] (2021). Ergotherapie_Nederland. https://info.ergotherapie.nl/Overzichtcoronadocumenten:%20Ergotherapie_Nederland.

[24] Gerards MHG, Slotegraaf AI, Verburg AC (2024). One-year evaluation of people recovering from COVID-19 receiving allied primary healthcare: A nationwide prospective cohort study. Ann Phys Rehabil Med.

[25] Slotegraaf AI, Gerards MHG, Verburg AC (2023). Evaluation of primary allied healthcare in patients recovering from covid-19: first results after six months follow-up in a dutch nationwide prospective cohort study. Respiratory Medicine.

[26] C-Support, Erasmusmc (2023). Long COVID onderzoek: 1 jaar.

[27] C-support, Erasmusmc (2023). Long COVID onderzoek.

[28] Ceban F, Ling S, Lui LMW (2022). Fatigue and cognitive impairment in Post-COVID-19 Syndrome: A systematic review and meta-analysis. Brain Behav Immun.

[29] Shanbehzadeh S, Tavahomi M, Zanjari N (2021). Physical and mental health complications post-COVID-19: Scoping review. J Psychosom Res.

[30] Brus I, Spronk I, Tieleman P (2022). De Invloed van Long COVID Op Gezondheid, Dagelijks Leven, Werk En Zorg. Meerjarig Long Covid Onderzoek: Rapport Jaar 1.

[31] Alizadeh N, Packer T, Chen Y-T (2023). What we know about fatigue self-management programs for people living with chronic conditions: A scoping review. Pat Educ Couns.

[32] Alizadeh N, Packer TL, Jaswal S (2024). Client Perceptions of the Individual Packer Managing Fatigue Program: A Mixed-Method Evaluation. *OTJR: Occupational Therapy Journal of Research*.

[33] Slotegraaf AI, de Kruif AJTCM, Agasi-Idenburg CS (2024). Understanding recovery of people recovering from COVID-19 receiving treatment from primary care allied health professionals: a mixed-methods study. Disabil Rehabil.

[34] Heaton-Shrestha C, Torrens-Burton A, Leggat F (2022). Co-designing personalised self-management support for people living with long Covid: The LISTEN protocol. PLoS One.

[35] Skivington K, Matthews L, Simpson SA (2021). A new framework for developing and evaluating complex interventions: update of Medical Research Council guidance. BMJ.

